# Continuous Indexing of Fibrosis (CIF): improving the assessment and classification of MPN patients

**DOI:** 10.1038/s41375-022-01773-0

**Published:** 2022-12-05

**Authors:** Hosuk Ryou, Korsuk Sirinukunwattana, Alan Aberdeen, Gillian Grindstaff, Bernadette J. Stolz, Helen Byrne, Heather A. Harrington, Nikolaos Sousos, Anna L. Godfrey, Claire N. Harrison, Bethan Psaila, Adam J. Mead, Gabrielle Rees, Gareth D. H. Turner, Jens Rittscher, Daniel Royston

**Affiliations:** 1 Nuffield Division of Clinical Laboratory Sciences, Radcliffe Department of Medicine, University of Oxford, Oxford, UK; 2 Institute of Biomedical Engineering (IBME), Department of Engineering Science, University of Oxford, Oxford, UK; 3 Big Data Institute/Li Ka Shing Centre for Health Information and Discovery, University of Oxford, Oxford, UK; 4 Ground Truth Labs, Oxford, UK; 5 Oxford NIHR Biomedical Research Centre, Oxford University Hospitals NHS Foundation Trust, Oxford, UK; 6 Department of Mathematics, University of California, Los Angeles, CA USA; 7 Mathematical Institute, University of Oxford, Oxford, UK; 8 Laboratory for Topology and Neuroscience, École Polytechnique Fédérale de Lausanne, Lausanne, Switzerland; 9 Ludwig Institute for Cancer Research, University of Oxford, Oxford, UK; 10 Wellcome Centre for Human Genetics, University of Oxford, Oxford, UK; 11 Department of Haematology, Oxford University Hospitals NHS Foundation Trust, Oxford, UK; 12 MRC Weatherall Institute of Molecular Medicine, Radcliffe Department of Medicine, University of Oxford, Oxford, UK; 13 Haematopathology & Oncology Diagnostics Service, Cambridge University Hospitals NHS Foundation Trust, Cambridge, UK; 14 Department of Haematology, Guy’s and St Thomas’ NHS Foundation Trust, London, UK; 15 Department of Pathology, Oxford University Hospitals NHS Foundation Trust, Oxford, UK

**Keywords:** Diagnosis, Myeloproliferative disease

## Abstract

The grading of fibrosis in myeloproliferative neoplasms (MPN) is an important component of disease classification, prognostication and monitoring. However, current fibrosis grading systems are only semi-quantitative and fail to fully capture sample heterogeneity. To improve the quantitation of reticulin fibrosis, we developed a machine learning approach using bone marrow trephine (BMT) samples (*n* = 107) from patients diagnosed with MPN or a reactive marrow. The resulting Continuous Indexing of Fibrosis (CIF) enhances the detection and monitoring of fibrosis within BMTs, and aids MPN subtyping. When combined with megakaryocyte feature analysis, CIF discriminates between the frequently challenging differential diagnosis of essential thrombocythemia (ET) and pre-fibrotic myelofibrosis with high predictive accuracy [area under the curve = 0.94]. CIF also shows promise in the identification of MPN patients at risk of disease progression; analysis of samples from 35 patients diagnosed with ET and enrolled in the Primary Thrombocythemia-1 trial identified features predictive of post-ET myelofibrosis (area under the curve = 0.77). In addition to these clinical applications, automated analysis of fibrosis has clear potential to further refine disease classification boundaries and inform future studies of the micro-environmental factors driving disease initiation and progression in MPN and other stem cell disorders.

## Introduction

Reticulin fibers are an important component of the bone marrow extracellular matrix (ECM) essential for the maintenance of hematopoiesis. In normal marrow, silver impregnation techniques highlight the reticulin substrate as a delicate network of thin, uniform fibers coursing through the intertrabecular spaces, with variable condensation around small blood vessels. While this organized reticulin meshwork is perturbed in several pathological conditions [1–3], the diagnostic and prognostic importance of abnormal reticulin is best characterized in the Philadelphia-negative myeloproliferative neoplasms (MPNs), a group of disorders in which acquired mutations in hematopoietic stem cells affect the MPL-JAK-STAT signaling pathway and drive excessive proliferation of ≥1 blood lineage [4–7]. Although the precise mechanisms driving marrow fibrosis remain poorly understood, the initiation and progression of fibrosis in MPNs reflects a pathological cytokine/chemokine-driven inflammatory response to clonal myeloproliferation, induced by neoplastic hematopoietic stem cells (HSC) [8–13].

The importance of fibrosis estimation in MPNs is embedded in the World Health Organization (WHO) classification scheme of the common MPNs: essential thrombocythemia (ET), polycythemia vera (PV), primary myelofibrosis (PMF) and pre-fibrotic primary myelofibrosis (pre-PMF) [14]. Fibrosis severity also has clinical implications in MPNs, with minor fibrosis (MF-1) in PV associated with inferior survival and more advanced fibrosis associated with a complex karyotype [15, 16]. In PMF, increasing fibrosis is associated with worsening hematological and clinical parameters and overall prognosis [17–19], with the presence of MF ≥2 identified as a significant risk factor in the MIPSS70 prognostic model that incorporates high-risk molecular mutations [20]. The latest version of the WHO fibrosis scoring system comprises four categories (MF-0–3) that attempt to encompass escalating deposition of reticulin fibers, fiber intersections, bundling of collagen and/or osteosclerosis. In addition to reticulin staining, a trichrome to detect collagen is also advised in cases of MF-2 and MF-3. These grade descriptions are qualitative and subjective, but several studies have demonstrated reasonable-to-good concordance between hematopathologists [21–24]. Nonetheless, the WHO grading scheme fails to comprehensively accommodate fibrotic heterogeneity within BMT specimens, specifying only that the final grade is determined by the highest grade present in ≥30% of the marrow area.

In response, we sought to develop an automated machine learning (ML) methodology to objectively quantify reticulin fibrosis using routinely prepared reticulin-stained BMT samples of normal/reactive and MPN samples. Manually annotated regions of fibrosis were used to create an initial ranked list of fibrosis severity in which uniform image tiles received a predicted fibrosis score between 0 and 1 (Continuous Index of Fibrosis [CIF]). The predicted scores of new, unseen tiles were then converted to a quantitative fibrosis map overlaid onto whole sample images. Analysis of MPN sample cohorts allowed us to capture the full spectrum and heterogeneity of fibrosis within established MPN and normal/reactive BMT samples. We hypothesized that such an approach would enhance the accuracy of fibrosis assessment in MPN samples, with implications for improved disease classification (particularly distinction of ET and pre-PMF) and refined disease monitoring. To assess the potential for improving disease prognostication, we also applied our methodology to a set of well-characterized ET patients with long-term clinical follow-up.

## Materials and methods

**Fig. 1:**
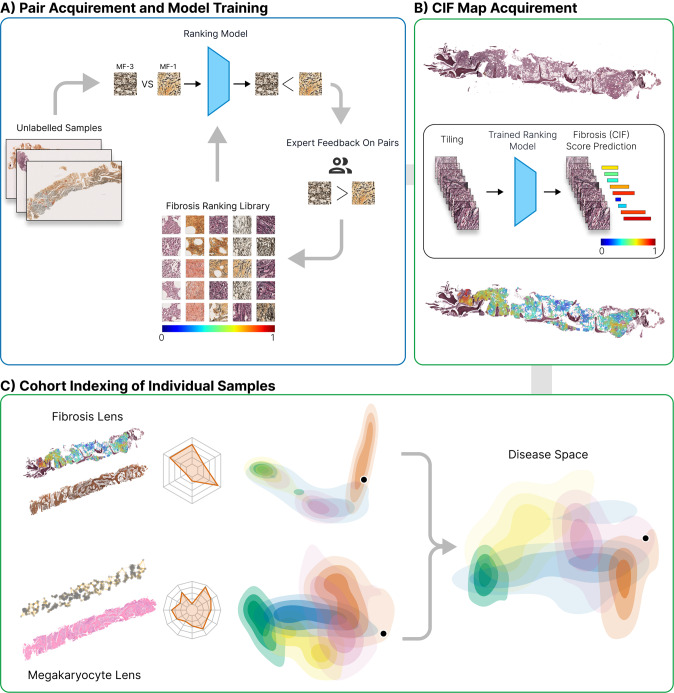
Overview of the computational steps for detection, quantitation and visualization of reticulin fibrosis in BMTs. **A** Image tiles are extracted from manually segmented areas of fibrosis and labeled with the corresponding grade. Extracted tiles are then used to train a ranking-CNN (convolutional neural network) model to output scores using a pairwise sample approach, with higher scores corresponding to more severe fibrosis. Based on the initial trained model, we adopted a human-in-the-loop approach for manual image ranking. **B** With the finalized model, a CIF map of each sample is acquired by predicting the scores of all extracted tiles and visualizing this as a color map superimposed upon the original reticulin-stained image. **C** From the predicted CIF scores and measurements of tile distribution, the fibrosis features are represented in two-dimensional disease space to allow comparison of MPN subtypes and indexing of individual patient BMTs against a sample library. Fibrosis features can then be combined with those of other BMT constituents (e.g., megakaryocytes) to refine the disease space.

An overview of the methodologies employed in this study is given in Fig. 1.

### Clinical samples

BMT samples fixed in 10% neutral buffered formalin prior to decalcification in 10% EDTA (48 h) were obtained from the archive of OUH NHS Foundation Trust. For inclusion, BMT sections had to be of sufficient size (≥5 intact intertrabecular spaces) and quality for conventional reporting, as determined by a specialist hematopathologist (DR). Whole slide scanned images (Hamamatsu NanoZoomer 2.0HT/40×/NDPI files or 3DHISTECH 250 Flash III Dx/40×/MRXS files) were prepared from 2–3 μm reticulin-stained (Gomori and Sweet) sections cut from formalin-fixed paraffin-embedded (FFPE) blocks. The data set comprised 107 diagnostic samples from patients who had received no disease-modifying treatment (36 ET, 19 PV, 23 MF, 17 pre-PMF, and 12 reactive/nonneoplastic), with “reactive” samples sourced from patients in whom there was no evidence of malignancy or persistent thrombocytosis. For two of the MF patients, sequential samples (5 BMTs each) taken over 12 or 23 months were also included. All patients diagnosed with MPN had been reviewed as part of a regional myeloid multidisciplinary meeting (MDM). A summary of the key patient characteristics is provided in Supplementary Table 1 and Supplementary Fig. 1. Additional MPN samples (35) were obtained from the Primary Thrombocythemia-1 (PT-1) trial cohort; a multicenter ET trial in which newly diagnosed and previously treated patients were recruited into one of three studies (previously published) depending on their risk of vascular complications [25–27]. This work was conducted as part of the INForMeD study (INvestigating the genetic and cellular basis of sporadic and Familial Myeloid Disorders; IRAS ID: 199833; REC reference: 16/LO/1376; PI: Prof AJ Mead), with all patients providing written informed consent.

### Automated identification of fibrosis and severity assessment

Reticulin staining employs silver impregnation to highlight reticulin fibers as black linear material. Minor variations in routine laboratory practice (including counterstaining and toning) impart a range of colors to bone and cellular elements. Digitized reticulin images were, therefore, converted into grayscale to prevent any color variation from adversely influencing our model’s performance [28, 29]. Two sets of BMT samples capturing the spectrum of marrow fibrosis were used for the training (39 samples) and validation (18 samples) steps of our model generation. For the initial training and validation stages, uniformly sized tiles (512 × 512 pixels [0.22 μm per pixel]) were extracted from manually segmented samples deemed suitable for fibrosis estimation. A deep learning model based on UNet [30] was used to assist in the segmentation and exclusion of bony trabeculae (Supplementary Table 2). For subsequent rounds of training and validation, a sliding window of 512 × 512 pixels with a stride of 256 pixels (Supplementary Fig. 2) was used to extract tiles that satisfied each of three criteria: fat regions account for <50% of the tile area; bone or bone fragments account for <1% of the tile area; and, blood vessels account for <10% of the tile area. We reasoned that this rule set maximized the analyzable area of each sample while adhering to the convention of restricting fibrosis grading to areas of hematopoiesis.

To accommodate a continuous spectrum of fibrosis severity within and between MF grades, we employed a Learning to Rank (LTR) strategy called RankNet to train a model that estimates sample fibrosis in the form of an ordered ranking of feature severity [31]. This RankNet approach was then combined with a Convolutional Neural Network (CNN) to build a Ranking-CNN model [30, 32, 33] (Supplementary Table 3). To determine the ground truth of analyzed images, a pairwise ranking strategy suitable for rapid and intuitive human review was adopted, with three specialist hematopathologists selecting the most severe of two candidate image regions using conventional WHO fibrosis criteria. (Please refer to Supplementary Fig. 3 for an overview of the initial tile pair acquisition and model training.) A normalized range of output scores between 0 and 1 was used as the reference of fibrosis severity, with scores (CIF scores) approaching 1 being the most fibrotic.

We adopted a human-in-the-loop approach for manual image ranking (Supplementary Fig. 4) as this minimized the number of pairwise image comparison tasks for each iteration of model training and validation (Supplementary Methods: Training of the ranking-CNN; Supplementary Table 4 and Supplementary Fig. 5).

### Image mapping of fibrosis severity and feature extraction

Generating fibrosis severity maps from our CIF model output scores is an efficient and intuitive method of visualizing fibrosis throughout a sample. To acquire these CIF maps, a sliding window of 512 × 512 pixels was used within the annotated region, with a stride of 256 pixels. To allow subsequent comparison between samples, three sets of features relating to the analyzed tiles extracted from each sample were used: average CIF score, tile distribution, and Shannon entropy of tile distribution. Shannon entropy (henceforth “heterogeneity”) captures the “unevenness” of tile scores, with tile distribution reflecting the extent to which particular CIF scores are enriched in each sample. As the output CIF scores from our model were continuous between 0 and 1, scores were divided into four “bins” that broadly correspond to WHO fibrosis grades MF-0, MF-1, MF-2 and MF-3. The difference in fibrosis between MPN subtypes was calculated via the Mann–Whitney–Wilcoxon test (non-parametric with no assumptions of the data distribution) where *P* value (*P*) < 0.05 is considered statistically significant.

### Topological data analysis of ET and pre-PMF samples

To interrogate the relationship between fibrotic foci within ET and pre-PMF, we employed topological data analysis (TDA), a relatively new field in computational mathematics that studies the shape and connectivity of data [34–36]. Persistent homology, a prominent and robust TDA algorithm [37], enabled us to explore the connectivity pattern of fibrotic foci across a continuous range of spatial scales within our samples. The input for this analysis was the CIF tile scores and the output was a multiscale summary of the spatial connectivity of the CIF scores called a barcode, a topological fingerprint generated using Python Ripser version 0.6.2 [38]. The barcode tracks the persistence and connectivity of fibrotic foci as they appear and evolve in the image at different threshold values of the CIF score. Quantitative properties of the barcode could then be used for subsequent analysis and classification. To distinguish ET from pre-PMF, a random forest classifier was applied in Python, using the package scikit-learn [39], with a classifier comprising 100 decision trees. The importance of individual features was assessed using Gini importance [40, 41]. For further details and a description of the topological statistics used for this analysis, please refer to Supplementary Methods: Topology data analysis.

## Results

### Estimation of BMT fibrosis severity using a ranked list approach

**Fig. 2:**
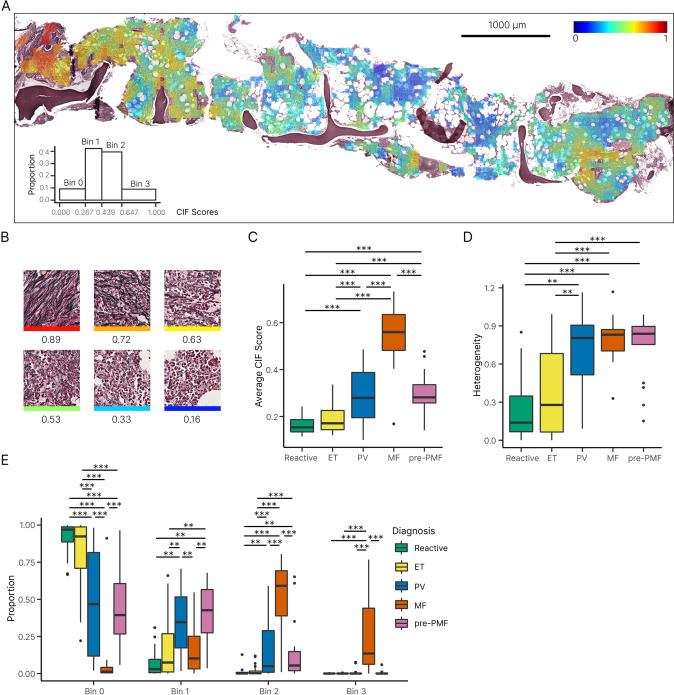
Distribution of tile CIF scores across MPN and reactive samples. **A** Example of a false-colored fibrosis heatmap, with CIF scores overlaid onto the original reticulin-stained BMT image [summary of tile distribution in the bottom left]. **B** Individual tiles receive a CIF score from 0 to 1 depending on the severity of reticulin fibrosis, with high scores assigned to tiles displaying increased fiber quantity, thickness and intersections. Boxplots of the **C** average CIF score and **D** heterogeneity of tile distribution for MPN and reactive samples. **E** Boxplot for the distribution of CIF scores (grouped into bins of increasing fibrosis) for MPN and reactive/normal samples. Results with *P* < 0.05 were considered statistically significant and are indicated by an asterisk (*0.01 < *P* ≤ 0.05, **1.00e–03 < *P* ≤ 0.01, ***1.00e–04 < *P* ≤ 1.00e–03). All *p* values are adjusted with Bonferroni correction. Confidence intervals of the median (CI) = 95%.

**Fig. 3:**
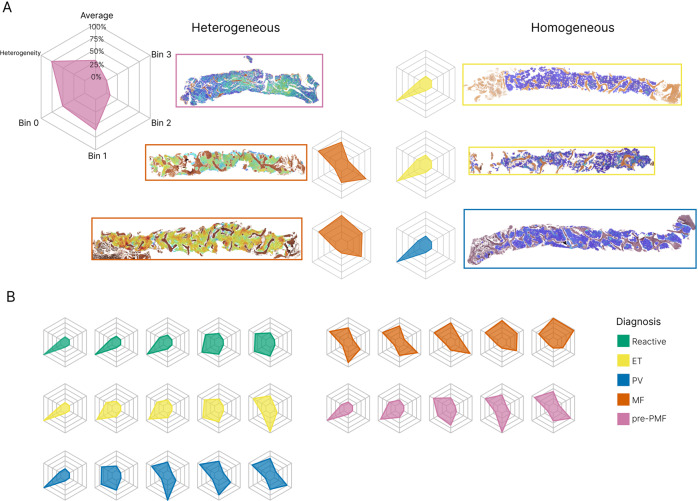
Variability of reticulin fibrosis within BMT samples. **A** Radar plots capture the average CIF tile score, tile distribution across the four bins, and heterogeneity of tile distribution. Examples of homogenous and heterogenous patterns of fibrosis are shown. **B** Examples of radar plots for reactive samples and each MPN subgroup.

We employed a human-in-the-loop methodology to efficiently build a ranked list of fibrosis severity comprising 11,448 image tiles extracted from reticulin-stained BMT sections. Following an initial round of pairwise ranking using tiles extracted from manually annotated whole slide-images, two subsequent rounds of manual ranking were fed back into the ML model for further training. The average manual ranking concordance by three hematopathologists after the first round of training and validation was high (88.40%; Supplementary Table 5). After three rounds of training and validation, our fibrosis ranking model achieved 93.99% accuracy (see Supplementary Tables 6 and 7 for the ranking performance within different image pairs and interobserver agreement). As expected, our model ranked highly fibrotic sample areas as those containing numerous thick reticulin fibers and bundles with frequent intersections (Fig. 2B and Supplementary Fig. 6). To better understand the output of the ranking model, we converted the normalized CIF tile scores to a color scale that could be superimposed upon a BMT image to generate a false-colored image (Figs. 2A and 3).

### Heterogeneity of reticulin fibrosis in BMT samples is associated with MPN subtype

To compare fibrosis quantitation between MPN subtypes and reactive samples, we determined average whole sample statistics that captured fibrosis severity and heterogeneity (Figs. 2C, D and 3). As expected, MF samples demonstrated significantly more fibrosis (higher average CIF score) than other MPN subtypes or the reactive/normal marrows. Equally expected was the finding of no significant difference between the average fibrosis scores in ET and reactive/normal samples; areas of minimal fibrosis amounting to MF-1 being well described in healthy marrow. In keeping with previous descriptions of patchy and variable fibrosis in PV, the average fibrosis score for PV was moderately higher than that of ET (PV 0.30 vs ET 0.19, *P* = 5E–4). Average fibrosis scores for PV and pre-PMF were identical (PV 0.30 vs pre-PMF 0.30, *P* = 0.94). Of particular interest, pre-PMF samples contained a significantly higher average CIF score than ET (pre-PMF 0.30 vs ET 0.19, *P* = 8E–5), despite meeting the diagnostic requirement of containing ≤ WHO grade MF-1 by conventional histological assessment. Given the importance of BMT histological assessment in distinguishing patients with ET and pre-PMF, this result suggested that our automated fibrosis analysis may have clinical utility in resolving this frequently challenging differential diagnosis.

In order to determine the distribution of the tile scores for each MPN subtype, we subdivided the tile CIF scores into four distinct bins that broadly correspond to each of the four established WHO fibrosis grade categories (Fig. 2E and Supplementary Table 8). As expected, MF cases accounted for almost all of the tiles assigned to bin 3, although less fibrotic/non-fibrotic tiles were also frequently encountered in MF samples. Also expected was the observation that ET samples predominantly comprised tiles from bins 0 and 1 (82.36% and 16.10%, respectively), consistent with fibrosis in ET seldom exceeding focal areas of conventional WHO grade MF-1 (Supplementary Table 9). The PV samples contained a fairly wide distribution of tiles from bins 0 to 2, with significantly more tile scores allocated to bin 1 than ET (PV 0.35 vs ET 0.16, *P* = 0.002) and bin 2 (PV 0.17 vs ET 0.02, *P* = 5E–4). Notably, samples of pre-PMF contained tile scores that were significantly different from those of ET. Despite being predominantly composed of tiles assigned to bins 0 and 1 (46.52% and 38.85%, respectively), tile scores assigned to bin 2 were significantly increased in the pre-PMF samples (pre-PMF 14.16% vs ET 1.51%, *P* = 2E–4), although areas of obvious WHO grade MF-2 (as determined by routine histological review) were absent from these samples in line with current WHO diagnostic criteria (Supplementary Table 9). Tile score distributions observed for the PV and pre-PMF samples were not significantly different. Of note, fibrosis heterogeneity did not appear to be simply correlated to average CIF scores, with no significant difference observed between the fibrosis heterogeneity of MF, PV and pre-PMF samples (MF 0.80 vs PV 0.69, *P* = 0.50; MF 0.80 vs pre-PMF 0.74, *P* = 0.96; PV 0.69 vs pre-PMF 0.74, *P* = 0.66).

These results revealed that a significant proportion of analyzed tiles with CIF scores assigned to bin 2 were not, in fact, derived from sample areas readily discernible by hematopathologists as equating to moderate/severe fibrosis amounting to WHO grade MF-2. This partly reflects the presence of microfoci or “hotspots” of advanced fibrosis that are either too small or too subtle to identify using conventional microscopy. Indeed, review of the CIF maps confirmed the presence of such microfibrotic hotspots throughout many MPN samples, most notably pre-PMF and PV.

### Cohort indexing of automated MPN fibrosis supports disease classification and assessment of disease progression

**Fig. 4:**
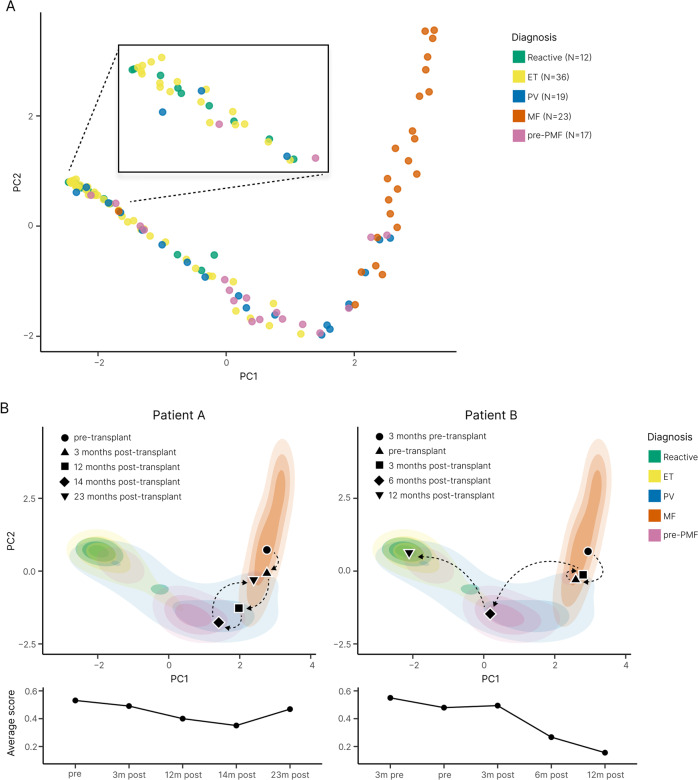
Disease cohort indexing discriminates between MPN samples and supports disease monitoring. **A** PCA plot of the abstract representations of sample fibrosis reveals the clustering of reactive cases and MPN subtypes. **B** Indexing the multivariable representation of fibrosis from individual patient samples to the PCA plot of the sample cohort enables monitoring of fibrotic progression. MF patients A and B underwent allogeneic bone marrow transplantation with subsequent marrow sampling to monitor disease response. Patient A demonstrated only modest improvement of marrow fibrosis post-transplant, with evidence of fibrotic relapse at 23 months (consistent with the results of clinical and laboratory disease monitoring). By contrast, Patient B demonstrated a profound reduction/normalization of marrow fibrosis within 12 months of transplantation.

To enhance the visualization of our automated analysis, we performed principal component analysis (PCA) to create an abstracted two-dimensional space that incorporates the average tile CIF score, tile distribution, and heterogeneity of tile distribution for our sample cohort (Fig. 4A). As expected, PCA demonstrated clear separation of MF from reactive/normal and ET samples, with cases of PV seen to overlap each region in PCA space. The distribution of the PV samples in PCA space did not appear to be strongly associated with the JAK2 V617F variant allele frequency (Supplementary Fig. 7). The relationship between driver mutation status and PCA distribution for the ET, MF and pre-MF samples is shown in Supplementary Fig. 8.

Notably, when ET and pre-PMF samples were directly compared, both appeared to aggregate in distinct regions of the PCA plot with only minor overlap. Based on the PCA feature representations, we trained a random forest classifier to distinguish ET (*n* = 36) from pre-PMF (*n* = 17) samples. In three-fold cross-validation (used to estimate the performance of a model by which data are split into three groups of approximately equal size) the classifier reached an area under the curve (AUC) of 0.71 for discriminating between these MPN subtypes. Of note, two pre-PMF samples were seen to overlap with the PCA space primarily occupied by samples of MF, despite meeting WHO morphological diagnostic criteria including an overall WHO fibrosis grade of ≤ MF-1.

In addition to allowing a simplified assessment of the distribution of fibrosis within a cohort of reactive and MPN samples, PCA analysis allows changes in marrow fibrosis to be objectively detected and intuitively appreciated across sequential samples. This is of particular value in the interpretation of BMTs obtained from patients undergoing repeated biopsy to monitor disease response or progression (Fig. 4B).

### Topological data analysis (TDA) of fibrotic features in ET and pre-PMF samples

**Fig. 5:**
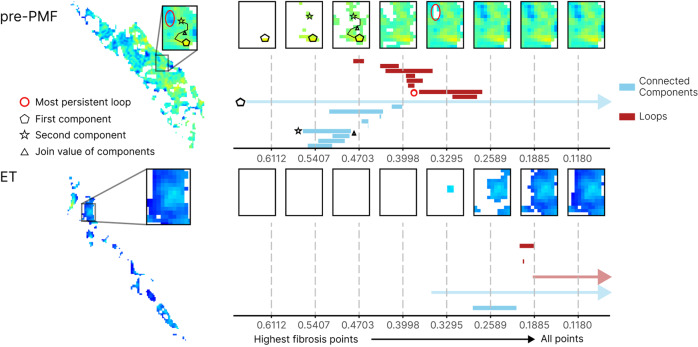
Topological data analysis (TDA) of fibrosis in ET and pre-PMF samples. Illustration of TDA for fibrotic foci in an ET and pre-PMF sample. Super-level set filtration on the images (left) allows computation of a topological fingerprint in the form of a barcode. The *x*-axis of the barcode corresponds to filtration values, i.e., fibrosis score thresholds, in the super-level set filtration. At high filtration values only the most fibrotic score pixels are included, with all points included at the lowest filtration values. The barcode in blue captures clusters or connected components of the hotspots (light blue bars) across the filtration; for high filtration values this corresponds to fibrosis hotspots which then merge with neighboring hotspots when all pixels between them are present in the filtration. Two examples of such hotspots in the filtration of a sample of pre-PMF are shown as a pentagon and a star, with the filtration value at which they merge highlighted by a triangle. In the barcode, we highlight the CIF values at which the hotspots are “born” in the corresponding bars with a pentagon and a star. When the pentagon and the star hotspots merge, the bar corresponding to the star hotspot ends (indicated by the triangle) and the now joint star-pentagon component continues to be monitored in the bar that corresponds to the pentagon hotspot. Infinite features, i.e. features that continue beyond the end of the filtration such as the clusters that consist of all fibrosis pixels in the sample once all pixels are included in the filtration, are represented as arrows extending beyond the *x-*axis. The barcode in red tracks one-dimensional holes (red bars) and their scale, also called persistence, in the images. The most persistent hole in the pre-PMF image is highlighted as a circle. We show the fibrosis score at which a hole appears (left endpoint) of the corresponding bar in the barcode with a circle.

Having identified significant differences in the average CIF tile score, tile distribution and heterogeneity between ET and pre-PMF, we sought to explore in more detail the fibrotic features associated with each subtype. We therefore extended our fibrotic feature analysis to include topological features as these have provided useful insight into other complex biomedical datasets [34–36, 42]. The identified topological descriptors were combined with the original fibrotic features to train a random forest classifier (Supplementary Fig. 9 and Supplementary Table 10) with improved performance (AUC = 0.82 [combined TDA + original fibrotic features] vs AUC = 0.70 [original fibrotic features]). These topological differences corresponded to the structure of the fibrotic foci, with pre-PMF samples appearing to have a greater number of fibrotic foci that were also more likely to be connected by paths with high CIF scores when compared to ET, implying a potential spatial relationship between areas of advancing fibrosis in pre-PMF (Fig. 5).

### Automated fibrosis analysis identifies patients at risk of fibrotic progression

**Fig. 6:**
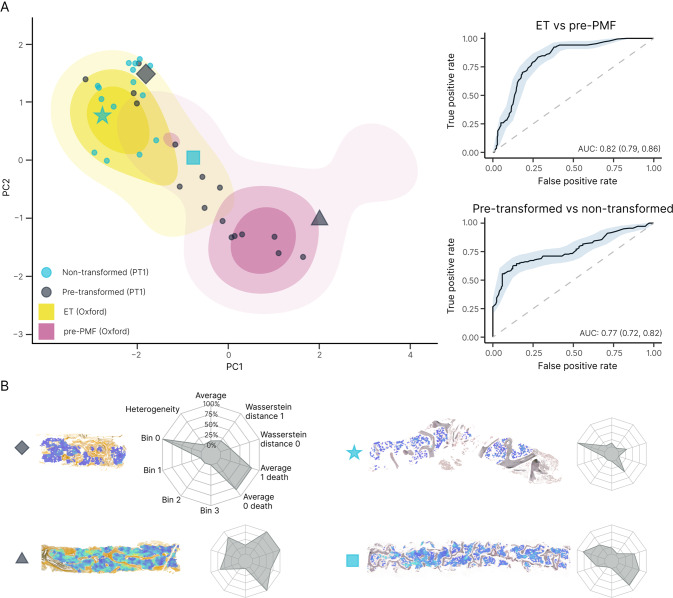
Combined fibrosis feature of ET, pre-PMF and PT-1 samples. **A** PCA plot of ET vs pre-PMF using fibrosis features (original + TDA) with overlay of pre-transformed and non-transformed cases of ET from the PT-1 cohort, along with the ROC curves for ET vs pre-PMF and pre-transformed vs non-transformed PT-1 samples. **B** Examples of the CIF maps and corresponding radar plots of the analyzed samples.

Given the evidence of good disease separation of ET and pre-PMF, we hypothesized that our approach may allow improved early detection of MPN patients at risk of progression to secondary myelofibrosis. To evaluate this, we interrogated the PT-1 clinical trial cohort for patients diagnosed with ET in whom there was diagnostic evidence of progression to secondary MF in the course of extended clinical follow-up. We identified 18 patients diagnosed with ET at trial enrollment in whom there was documented evidence of subsequent progression to post-ET myelofibrosis (median days to progression = 2356), and for whom we had access to analyzable pre-transformed reticulin-stained sections. As an internal control group, we identified 17 PT-1 trial participants in whom there was no diagnostic evidence of progression over a comparable or longer period of clinical follow-up (median follow-up = 4339 days). When indexed to the PCA plot derived from our locally sourced sample cohort (incorporating TDA), the PT-1 ET samples from non-transforming patients aggregated in the expected PCA space (Fig. 6A). By contrast, around half (9/17) of the subsequently transformed PT-1 ET samples were seen to aggregate in the PCA space corresponding to cases of pre-PMF from our local cohort. A random forest classifier trained to discriminate between patients who did or did not subsequently transform to post-ET myelofibrosis achieved an AUC of 0.77 (Fig. 6A and Supplementary Table 11).

### Integration of fibrotic and megakaryocytic features in MPN morphological assessment

**Fig. 7:**
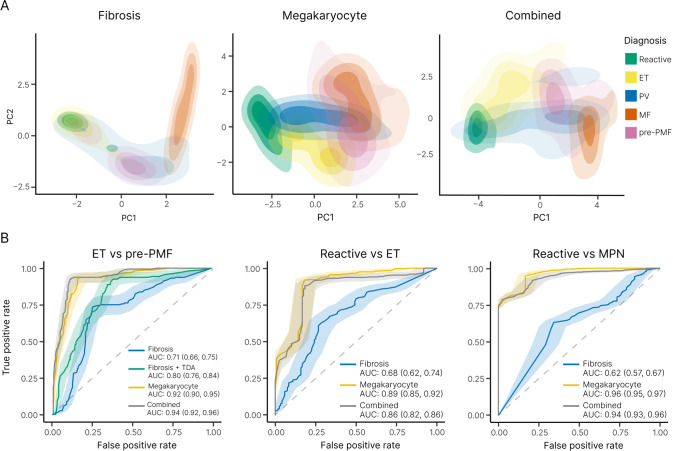
Combining fibrosis and megakaryocyte feature analysis improves the discrimination of MPN subtypes. **A** PCA plots of the fibrosis features, megakaryocyte features and combined features (fibrosis + megakaryocyte) reveal clustering of reactive samples and MPN subtypes. **B** Corresponding ROC curves (ET vs pre-PMF, reactive vs ET and reactive vs MPN) demonstrate the utility of combining fibrosis and megakaryocyte feature analysis in the assessment of MPNs, particularly for the discrimination of ET and pre-PMF samples (which includes TDA analysis of fibrotic features). For the purpose of comparison between fibrosis and megakaryocyte analyses for the ROC curve calculations, only samples for which both reticulin and H&E stains are available have been used (Reactive = 12, ET = 32, PV = 17, MF = 22, pre-PMF = 17).

Notwithstanding the diagnostic and prognostic potential of automated reticulin analysis, reticulin fibrosis is only one of several BMT features to be considered in the routine histological evaluation of MPNs. Indeed, in previous work employing ML to analyze megakaryocyte morphology and topology in BMTs we highlighted the potential of improved megakaryocyte analysis in the diagnosis and classification of MPNs [43]. Given the importance of both reticulin fibrosis and megakaryocyte analysis in MPN assessment, we sought to integrate both features using PCA in an attempt to improve the morphological resolution of MPN subtypes. When combined with our previous megakaryocyte feature PCA, fibrotic feature analysis demonstrated improved discrimination of ET and pre-PMF samples (AUC = 0.94 [megakaryocyte and fibrotic features] vs AUC = 0.92 [megakaryocyte features alone]) (Fig. 7B and Supplementary Table 10). By contrast, inclusion of fibrotic feature analysis did not enhance the discrimination of reactive and MPN (all subtypes) using megakaryocyte features (AUC = 0.94 [megakaryocyte and fibrotic features] vs AUC = 0.96 [megakaryocyte features alone]) (Fig. 7B and Supplementary Table 12), or the discrimination of reactive/normal and ET (AUC = 0.86 [megakaryocyte and fibrotic features] vs AUC = 0.89 [megakaryocyte features alone]) (Fig. 7B and Supplementary Table 13). This likely reflects the presence of variable amounts of minor fibrosis frequently encountered in healthy marrows, with significant feature overlap of non-fibrotic or mildly fibrotic MPN samples (Figs. 4A and 7A).

## Discussion

Here we describe a set of computational methods designed to systematically capture the key morphological characteristics of marrow fibrosis and associate them with particular MPN subtypes. This incorporates a platform that combines intuitive manual image-handling tools with support from an ML model, thereby aiding hematopathologists in the efficient ranking of marrow fibrosis severity. Our approaches remove dependency on a qualitative fibrosis grading system and have significant potential to assist hematopathologists in the morphological assessment of MPN patients, particularly in the challenging differential diagnosis of ET and pre-PMF.

Using TDA we demonstrate that microfoci of advanced fibrosis are a recurrent feature of pre-PMF, but are seldom encountered in ET. Detection and quantitation of these fibrotic microfoci is well beyond the scope of conventional histological assessment, and is not captured in current fibrosis grading classifications [14, 21]. The clinical significance of this finding is supported by our retrospective analysis of samples obtained as part of the PT-1 clinical trial of patients diagnosed with ET and receiving long-term follow-up. TDA identified fibrotic features, similar to those observed in pre-PMF patients, in over half of those ET patients (for whom slides were available) who subsequently progressed to post-ET myelofibrosis while enrolled on trial. Of note, patients eligible for PT-1 trial entry from 1997 to 2012 met the Polycythemia Study Group Diagnostic criteria for ET, before widespread recognition of pre-PMF as a diagnostic category and formal adoption by the WHO in 2016 [25–27]. This raises the possibility that at least a proportion of the ET patients subsequently transforming to secondary myelofibrosis might have had disease more in keeping with pre-PMF. Prospective studies determining the power of automated fibrosis assessment to predict myelofibrotic progression in ET and pre-PMF classified using the latest WHO criteria are clearly indicated.

In addition to direct clinical application, objectively monitoring and quantitating fibrosis in BMTs is ideally suited for studies evaluating the effect of current therapies on disease progression in MPN. The outputs are also well suited for integration into future clinical trial designs evaluating novel therapeutic targets/drug candidates [44, 45]. Without such approaches, incorporating marrow fibrosis assessment into robust clinical endpoints for the investigation of disease-modifying agents in myelofibrosis will remain challenging.

Our identification and description of fibrotic microfoci and related topological features within pre-PMF, and their association with fibrotic progression in ET, raises important questions about the factors driving early microfocal stromal fibrosis within the marrow. Recent evidence from murine and human studies suggests that mal-differentiation of mesenchymal stem cells (MSC), driven by neoplastic HSCs and their inflammatory microenvironment, are a major determinant of distinct pre-fibrotic and fibrotic phases of disease [10, 46]. The extent to which this process of stromal reprogramming is responsible for the microfoci of fibrosis identified in our current work clearly warrants further investigation. Moreover, the extent to which such early (potentially reversible) fibrotic foci may be important for widespread pathological changes in the surrounding marrow tissue, terminating in generalized marrow fibrosis, is also unclear. Intriguingly, analysis of the topological features embedded within our fibrosis data revealed not only increased numbers of fibrotic microfoci in pre-PMF samples when compared to ET, but also suggests that these fibrotic hotspots are spatially related, possibly reflecting local conditioning of the surrounding stromal tissue that predispose to further foci of early fibrosis development. This model of microfocal fibrotic progression in MPNs is entirely consistent with the growing body of evidence pointing to early HSC-driven abnormalities of the stem cell niche driving highly localized changes in the tissue microenvironment [8, 9, 47].

Statistical descriptions of bone marrow morphological features using enhanced image analysis techniques have only recently been described, and application to fibrosis complements our recent work describing megakaryocyte features in MPNs [43, 48]. Of note, while the specific ML strategies employed for detecting and quantitating fibrosis in the form of a continuous score (CIF score) are distinct from those previously employed in our megakaryocyte analysis, they draw upon shared technical and infrastructural processes and deliver outputs that are readily integrated into shared analytical workflows [49]. Indeed, we demonstrate how combining the morphological and topological features of fibrosis and megakaryocytes employed in conventional MPN diagnosis can be used to explore and refine our current understanding of disease boundaries. We recognize that additional cellular and stromal morphological features are important in MPNs, particularly cellular changes in non-megakaryocytic lineages and abnormalities of collagen deposition and bone. However, such features are in turn well suited to novel ML approaches. It is also acknowledged that the methods advanced in this work to refine the histological assessment of MPNs represent only one component of the integrated evaluation of myeloid diseases endorsed by the latest iteration of the WHO classification scheme. Further studies, including clinical trials, to evaluate the additional prognostic value of the ML features presented here are now indicated.

Fibrosis has long been recognized as an important pathological feature in diverse diseases affecting several organ systems [50], with ML approaches for fibrosis quantitation particularly well described in liver disease [51, 52]. Our strategy of refining the topological features of fibrosis in the context of curated patient cohorts and combining them with additional histological features is novel, and has significant potential for rapid translation into other organ systems.

## Supplementary information

Supplementary material

## Data Availability

The computer code and datasets generated during this study will be made available by the corresponding author upon request, in agreement with Cancer Research UK’s data sharing guidelines and after review by CRUK-Cancer Research Horizons.
