# Correction: Continuous Indexing of Fibrosis (CIF): improving the assessment and classification of MPN patients

**DOI:** 10.1038/s41375-023-01807-1

**Published:** 2023-01-12

**Authors:** Hosuk Ryou, Korsuk Sirinukunwattana, Alan Aberdeen, Gillian Grindstaff, Bernadette J. Stolz, Helen Byrne, Heather A. Harrington, Nikolaos Sousos, Anna L. Godfrey, Claire N. Harrison, Bethan Psaila, Adam J. Mead, Gabrielle Rees, Gareth D. H. Turner, Jens Rittscher, Daniel Royston

**Affiliations:** 1 Nuffield Division of Clinical Laboratory Sciences, Radcliffe Department of Medicine, University of Oxford, Oxford, UK; 2 Institute of Biomedical Engineering (IBME), Department of Engineering Science, University of Oxford, Oxford, UK; 3 Big Data Institute/Li Ka Shing Centre for Health Information and Discovery, University of Oxford, Oxford, UK; 4 Ground Truth Labs, Oxford, UK; 5 Oxford NIHR Biomedical Research Centre, Oxford University Hospitals NHS Foundation Trust, Oxford, UK; 6 Department of Mathematics, University of California, Los Angeles, CA USA; 7 Mathematical Institute, University of Oxford, Oxford, UK; 8 Laboratory for Topology and Neuroscience, École Polytechnique Fédérale de Lausanne, Lausanne, Switzerland; 9 Ludwig Institute for Cancer Research, University of Oxford, Oxford, UK; 10 Wellcome Centre for Human Genetics, University of Oxford, Oxford, UK; 11 Department of Haematology, Oxford University Hospitals NHS Foundation Trust, Oxford, UK; 12 MRC Weatherall Institute of Molecular Medicine, Radcliffe Department of Medicine, University of Oxford, Oxford, UK; 13 Haematopathology & Oncology Diagnostics Service, Cambridge University Hospitals NHS Foundation Trust, Cambridge, UK; 14 Department of Haematology, Guy’s and St Thomas’ NHS Foundation Trust, London, UK; 15 Department of Pathology, Oxford University Hospitals NHS Foundation Trust, Oxford, UK

**Keywords:** Diagnosis, Myeloproliferative disease

Correction to: *Leukemia* 10.1038/s41375-022-01773-0, published online 05 December 2022

In this article the affiliation details for Helen Byrne and Heather Harrington were incorrectly given as Department of Mathematics, University of California, Los Angeles, CA, USA but should have been Mathematical Institute, University of Oxford, Oxford, UK.

The original article has been corrected.

